# Elevated sclerostin levels in cerebrospinal fluid are associated with cognitive impairment in the Alzheimer's disease continuum

**DOI:** 10.1002/dad2.70417

**Published:** 2026-06-30

**Authors:** Patrizia Pignataro, Manuela Dicarlo, Daniele Urso, Chiara Zecca, Graziana Colaianni, Maria Teresa Dell'Abate, Davide Vilella, Francesco Borlizzi, Clelia Suriano, Angela Oranger, Silvia Colucci, Maria Grano, Giancarlo Logroscino

**Affiliations:** ^1^ Department of Translational Biomedicine and Neuroscience (DiBraiN) University of Bari “A. Moro,” Bari Italy; ^2^ Department of Precision and Regenerative Medicine and Ionian Area (DiMePRe‐J) University of Bari “A. Moro,” Bari Italy; ^3^ Center for Neurodegenerative Diseases and the Aging Brain University of Bari “A. Moro” at “Pia Fondazione Card G. Panico” Hospital Tricase Italy

**Keywords:** Alzheimer's disease, dementia, mild cognitive impairment, neurodegeneration, sclerostin

## Abstract

**Introduction:**

Bone‐derived sclerostin impairs cognitive function in both patients with Alzheimer's disease (AD) and mouse models of AD. For the first time, we explored the correlation of sclerostin with neurocognitive domains in the AD continuum.

**Methods:**

Sclerostin levels were measured in the cerebrospinal fluid (CSF) of a cohort of individuals with biological evidence of AD pathology, including patients with AD dementia, mild cognitive impairment due to AD (MCI), and subjective memory complaints (SMC). Correlations were examined between sclerostin and domain‐specific cognitive scores.

**Results:**

CSF sclerostin correlated with overall cognitive function, as well as with all cognitive domains impaired in AD. Of note, MCI patients with high CSF sclerostin levels displayed a worse overall and domain‐specific neurocognitive performance.

**Discussion:**

Our results evidenced the negative impact of sclerostin on all the cognitive domains predominantly in prodromal AD, suggesting its potential role as a biomarker of cognitive decline in the early stages of the disease.

## Highlights

- We explored the association between sclerostin and cognition in Alzheimer's disease (AD) staging.
- Cerebrospinal fluid (CSF) sclerostin correlated with all the cognitive domains impaired in AD.
- Patients with mild cognitive impairment with high CSF sclerostin showed worse cognitive performance.
- Sclerostin could be a biomarker of cognitive decline in the early stages of AD.

## 1 BACKGROUND

Alzheimer's disease (AD) is a neurodegenerative disorder clinically characterized by the progressive decline in cognitive function.^1^ The impairment of episodic memory—that is, the capacity to learn and maintain new information—is typically the initial symptom shared by most patients with AD.^2^ However, as AD progresses over time, several other cognitive domains, such as executive function, language, and spatial ability, are detrimentally impaired so that individuals are no longer capable of living independently.^2^

Despite the fact that early intervention could be helpful to delay disease progression before irreversible brain damage occurs, the identification of cognitive decline in subjects with very mild symptoms of cognitive dysfunction is still a crucial challenge.^3^

In the last years, an increasing number of studies have shown an intriguing association between AD and age‐related bone diseases, such as osteoporosis.^4^ Indeed, several meta‐analyses showed an increased incidence of cognitive impairment in patients with osteoporosis and parallel clinical studies have shown that patients with dementia were more susceptible to fractures.^5^ In‐depth investigations have unveiled the existence of a complex bidirectional communication between brain and bone in AD with several bone‐derived factors able to influence brain functions upon crossing the blood–brain barrier (BBB).^4^ Osteocalcin (OCN), lipocalin‐2, fibroblast growth factor 23 (FGF23), osteoprotegerin (OPG), and osteopontin (OPN) are some examples of factors produced by bone cells whose effects on the brain have been explored in the context of AD.^4^ Although these studies are still in their infancy, the first evidence suggested a neuroprotective effect of OCN against AD,^6^ while both FGF23 and OPN seem to be involved in AD pathophysiology.^7^, ^8^ Controversial results have emerged from studies conducted on lipocalin‐2 and OPG and will require more extensive investigations.^9^

Very recently, interesting findings emerged on the role of sclerostin, a glycoprotein mainly produced by osteocytes, in AD pathogenesis.^10^ Indeed, it was observed that sclerostin impaired the cognitive function in aged mice and in mouse models of AD by increasing amyloid beta (Aβ) deposition.^10^ Moreover, in the same study, it was demonstrated that there is a correlation between high serum sclerostin levels and cognitive impairment in cohorts of old subjects and patients with AD.^10^

However, investigation conducted to date has not focused on the association between sclerostin and the main cognitive domains in AD staging. To overcome this, in this study we extended our previous findings showing the relationship between cerebrospinal fluid (CSF) sclerostin and AD biomarkers^11^ to cognitive outcomes, investigating for the first time the association between this osteokine and cognitive performance in the AD continuum. In the cohort of our previous study including patients with AD dementia, mild cognitive impairment (MCI) due to AD, and subjective memory complaints (SMC) biologically defined according to the amyloid/tau/neurodegeneration (ATN) research framework,^12^, ^13^ we correlated CSF sclerostin levels with the scores of an extensive battery of neuropsychological tests that covered a wide range of cognitive domains, such as memory, executive function, attention, visuospatial abilities, and language.

## 2 METHODS

### 2.1 Participants

This study was conducted in a cohort of 146 participants previously described in our research on CSF sclerostin and AD biomarkers,^11^including individuals with diagnosis of AD dementia (*n* = 82), MCI (*n* = 44) due to AD, and SMC (*n* = 20). After enrollment at the Center for Neurodegenerative Diseases and the Aging Brain of the University of Study of Bari “Aldo Moro” at Pia Fondazione “Card. Panico” Hospital (Tricase), each patient underwent a multidisciplinary examination that included neurological and neuropsychological tests, nutritional assessment, 3T magnetic resonance imaging, standard laboratory tests, and lumbar puncture for CSF biomarker analysis. For AD dementia, the diagnosis was executed using the standard diagnostic criteria for dementia (Diagnostic and Statistical Manual of Mental Disorders, 5th edition [DSM‐V])^14^ and the National Institute on Aging–Alzheimer's Association (NIA‐AA) criteria.^12^, ^13^ For the prodromal stage of AD, that is, MCI due to AD, the diagnosis was based on the following DSM‐V criteria: (1) change in cognitive abilities reported by patient, informant, or clinician; (2) cognitive impairment in one or more of the following domains: memory, executive function, attention, visuospatial abilities, and language, confirmed by neuropsychological assessment; (3) preserved daily activities; and (4) absence of dementia.

**RESEARCH IN CONTEXT**

1. **Systematic review**: Literature regarding the role of bone‐derived sclerostin on cognitive impairment in Alzheimer's disease (AD) was reviewed using traditional search engines (e.g., PubMed). Sclerostin impairs cognition in AD patients and in mouse models of the disease; however, the association between the osteokine and the cognitive domains has not been explored in AD staging.
2. **Interpretation**: Our findings evidenced the correlation between cerebrospinal fluid sclerostin and all the cognitive domains impaired in AD, including memory, executive function, attention, visuospatial abilities, and language in a cohort of patients with biological evidence of AD pathology.
3. **Future directions**: The promising results of our cross‐sectional study should be confirmed by future longitudinal investigations on this topic. In addition, further research is needed to elucidate how sclerostin may be linked to structural and functional changes underlying cognitive impairment in AD.

For the SMC group, which included patients without significant impairment in cognition and in their daily living activities, the diagnosis was based on the following criteria: (1) persistent memory and cognitive function decline compared to a previously normal state and not induced by an acute event; and (2) normal cognitive performance confirmed by standardized psychometric tests used to classify MCI due to AD or prodromal AD.^15^ To rate cognitive impairment severity, the Clinical Dementia Rating (CDR) scale and CDR Sum of Boxes (CDR‐SOB) were used.

For the biological classification according to the NIA‐AA ATN framework,^12^, ^13^ all patients underwent a lumbar puncture to assess CSF AD biomarkers, that is, Aβ42, total tau (t‐tau), and phosphorylated tau (p‐tau)181. All patients with AD and MCI due to AD had evidence of AD pathology,^12^, ^13^ and were therefore classified as biologically AD positive. In contrast, all participants with SMC showed a negative AD biomarker profile (A–/T–/N–) according to the ATN classification system.^12^, ^13^ This study was approved by the local ethics committee (ASL Lecce, protocol no. 6, May 25, 2017) in accordance with the Declaration of Helsinki.^16^ Written informed consent was obtained from all patients. The study participants were selected for their clinical diagnosis, without age, sex, or race discrimination. Patients were carefully recruited to ensure sex balance and any form of discrimination.

### 2.2 Cognitive assessment

All patients were subjected to an extensive cognitive assessment comprising a total of 18 neuropsychological tests, including three screening tests and other tests that covered at least two cognitive domains. Table S1 in supporting information presents the details of the neuropsychological tests and cognitive domains.

Initially, the following screening tests were administered: the Mini‐Mental State Examination (MMSE) for overall cognitive function, the Frontal Assessment Battery (FAB) for frontal lobe functions, and the Clock Drawing Test (CDT) for executive and visuospatial abilities.^17^, ^18^, ^19^

To examine the individual domains the following tests were used: the Rey Auditory Verbal Learning Test (RAVLT) and the Rey–Osterrieth Complex Figure (ROCF) for memory,^20^, ^21^the Digit Span Backward (DS‐B), the Verbal Fluency Test (VFT)–phonemic subtest, the Trail Making Test Part B (TMT‐B), and the Stroop Color and Word Test (SCWT) to assess executive function,^22^, ^23^, ^24^ the Digit Span Forward (DS‐F) and the Trail Making Test Part A (TMT‐A) to test attention,^22^, ^23^ the Italian Figure Copy Test and the “Incomplete Letters” subtest of the Visual Object and Space Perception Battery (VOSP) for visuospatial abilities,^25^, ^26^ and the Boston Naming Test (BNT‐15 items) and the VFT–semantic to examine language.^27^

### 2.3 CSF sample collection and storage

All patients enrolled in the study underwent lumbar punctures, according to standard procedures, to collect CSF samples. After centrifugation at 2000 x g for 10 minutes at room temperature, samples were aliquoted and stored at −80°C until their use.

### 2.4 Assessment of CSF AD biomarkers

The concentrations of Aβ42, t‐tau, and p‐tau181 were determined in CSF patient samples using a chemiluminescent immunoassay (CLEIA; Lumipulse G Aβ1–42, Lumipulse G Total Tau, Lumipulse G pTau181, Fujirebio Europe N.V.) on a fully automated platform (Lumipulse G600II, Fujirebio Europe N.V.). All the tests were performed in accordance with the manufacturer's instructions.

For the interpretation of CSF biomarker results, the following cut‐off values were applied: Aβ42 > 599 pg/mL, t‐tau < 342 pg/mL, and p‐tau181 < 57 pg/mL. According to established diagnostic criteria for AD,^28^ a CSF biomarker profile was considered indicative of AD when Aβ42 levels were below the cut‐off, together with t‐tau and/or p‐tau181 levels above their respective thresholds. In cases with borderline amyloid levels, the p‐tau/Aβ42 ratio was also evaluated to further support the interpretation of amyloid status.^29^

### 2.5 CSF sclerostin assay

Sclerostin concentrations in CSF samples were measured by enzyme‐linked immunosorbent assay (ELISA) using the Human SOST/Sclerostin Quantikine kit (DSST00, R&D Systems, Inc.). The assay had a minimum detectable dose of 3.8 pg/mL and a measurement range of 31.3 to 2000 pg/mL. According to the manufacturer's instructions, all CSF samples and standards were assayed repeatedly and in duplicate. The plate absorbance was read at 450 nm in a plate reader (Eon, BioTek). Readings at 540 nm were subtracted to correct for any optical plate imperfections. Results were expressed in pg/mL. Sclerostin concentrations below the lower limit of quantification were extrapolated from the standard curve by the ELISA software. Given their proximity to the assay detection limits, these measurements were considered semi‐quantitative estimates rather than fully reliable absolute concentrations. Samples with signals below the zero standard were considered by the software as undetectable.

### 2.6 Statistical analysis

Data analysis was performed using GraphPad Prism software (version 9.5.0; GraphPad Software) and JASP software (version 0.19.3; JASP Team, 2025). Categorical variables, such as sex, were analyzed using Pearson chi‐squared tests. For continuous variables, data distribution was assessed by the Shapiro–Wilk test. Table S2 in supporting information presents the results of this analysis. For two‐group comparisons, the two‐tailed unpaired Student test and the Mann–Whitney test were used for normally and non‐normally distributed data, respectively. Analysis of variance (ANOVA) and the Tukey post hoc test and Kruskal–Wallis and Dunn post hoc test were used for three‐group comparisons of normally and non‐normally distributed data, respectively. Given the non‐normal distribution of CSF sclerostin levels in all patients, and in the female and male subgroups (reported in Table S2), all correlations were evaluated by the Spearman correlation test, while the Spearman partial correlation test was used to adjust correlations for age, sex, and education. For the cognitive domains, in Spearman correlation analyses, *p* values were adjusted for multiple comparisons using the Holm sequential Bonferroni method (Bonferroni–Holm correction) to prevent false positive conclusions (Type I errors). Differences between groups and correlations were considered significant when *p* < 0.05.

Receiver operating characteristic (ROC) curve analysis was used to evaluate the ability of CSF sclerostin to discriminate cognitively normal subjects (SMC) from cognitively impaired patients (MCI due to AD and AD). ROC curve analyses were also performed separately for pairwise comparisons between SMC and MCI, SMC and AD, and MCI and AD groups. For each ROC curve, the area under the curve (AUC), its 95% confidence interval (CI), and the *p* value for testing the null hypothesis of AUC = 0.5 were calculated to assess CSF sclerostin discriminatory performance.

## 3 RESULTS

### 3.1 Demographic and clinical information of the study cohort

The participants enrolled in this study consisted of patients with AD dementia (*n* = 82), MCI due to AD (*n* = 44), and SMC (*n* = 20) with an equal number of women and men (Pearson chi‐squared test, *p* = 0.367). Patients with AD dementia showed a higher mean age than those with SMC (*p* = 0.006), while their education level was significantly lower than patients with SMC (*p* = 0.026). Furthermore, the mean CDR and CDR‐SOB scores were significantly higher in patients with AD compared to MCI due to AD and SMC (*p* < 0.0001 for both).

The results of the neuropsychological examination showed significant differences among patients with SMC, MCI due to AD, and AD. As presented in Table S3 in supporting information, the performance of patients with AD was significantly worse than that of MCI due to AD or SMC for all the cognitive domains.

### 3.2 CSF sclerostin levels in patients with AD, MCI, and SMC

As reported in our previous study,^11^ the levels of sclerostin in the CSF were significantly different in the clinical stages of AD. Indeed, patients with AD dementia showed higher CSF sclerostin concentration than patients with MCI due to AD (6.85 ± 15.24 vs. 2.31 ± 6.89 pg/ml; *p* = 0.032). Similarly, patients with AD displayed higher CSF sclerostin levels also compared to those with SMC (6.85 ± 15.24 pg/ml vs. not detectable; *p* = 0.001).

CSF sclerostin levels discriminated patients with SMC from those who were cognitively impaired (MCI due to AD + AD) with an AUC of 0.671 (95% CI: 0.568–0.773, *p* = 0.014). In pairwise analyses, CSF sclerostin did not significantly discriminate between SMC and MCI (AUC = 0.602, 95% CI: 0.462–0.743, *p* = 0.192), whereas it significantly discriminated between SMC and AD (AUC = 0.707, 95% CI: 0.604–0.811, *p* = 0.004). Discrimination between MCI and AD was weak but statistically significant (AUC = 0.612, 95% CI: 0.512–0.712, *p* = 0.038; Figure S1 in supporting information).

The association of CSF sclerostin with age and education showed a positive correlation with age (*r* = 0.243, *p* = 0.0032) and a negative trend with education (*r* = −0.148, *p* = 0.075).

### 3.3 Correlation between CSF sclerostin and cognition

For the screening tests (Figure S2 in supporting information), the statistical analyses evidenced negative correlations between CSF sclerostin levels and MMSE (*r* = −0.304; *p* = 0.0003), FAB (*r* = −0.358; *p* < 0.0001), and CDT scores (*r* = −0.257; *p* = 0.004), indicating the association between higher CSF sclerostin levels and worse global cognitive measures. Moreover, upon categorizing all patients based on their MMSE score, we found that CSF sclerostin levels were significantly higher in patients with moderate cognitive impairment (MMSE score: 11–20) compared to subjects with normal cognition (MMSE score: 25–30; *p* = 0.0018; Figure S3 in supporting information).

Also, memory functions worsened with increasing CSF sclerostin levels; indeed, we observed negative correlations between sclerostin and RAVLT immediate (*r* = −0.341; *p* = 0.0001; Bonferroni–Holm corrected *p* = 0.0004), and delayed scores (*r* = −0.328; *p* = 0.0002; corrected *p* = 0.0006). On ROCF performance, we noticed a negative correlation with CSF sclerostin (*r* = −0.430; *p* = 0.0028; corrected *p* = 0.0056; Figure S4 in supporting information).

For executive function, higher CSF sclerostin levels were associated with poorer performance, as reflected by the negative correlations with DS‐B (*r* = −0.262; *p* = 0.004; corrected *p* = 0.016), and VFT–phonemic scores (*r* = −0.189; *p* = 0.032; corrected *p* = 0.064) and the positive correlations with TMT‐B scores (*r* = 0.373; *p* = 0.0016; corrected *p* = 0.008) and SCWT Errors (*r* = 0.258; *p* = 0.009; corrected *p* = 0.027; Figure S5 in supporting information).

For attention, we discovered a negative correlation between CSF sclerostin and DS‐F scores (*r* = −0.198; *p* = 0.030; corrected *p* = 0.030), and a positive correlation with TMT‐A scores (*r* = 0.264; *p* = 0.006; corrected *p* = 0.012), indicating worse attentional performance in patients with higher sclerostin levels (Figure S6 in supporting information).

For visuospatial abilities, CSF sclerostin was negatively correlated with copy figure (*r* = −0.288; *p* = 0.003; corrected *p* = 0.003), and VOSP Incomplete letters subtest scores (*r* = −0.309; *p* = 0.0007; corrected *p* = 0.0014; Figure S7 in supporting information).

For language, CSF sclerostin was negatively correlated with both BNT‐15 (*r* = −0.192; *p* = 0.0295; corrected *p* = 0.0295), and VFT–semantic scores (*r* = −0.264; *p* = 0.0032; corrected *p* = 0.0064; Figure S8 in supporting information).

After adjusting for some covariates (age, sex, and education), we observed that all the correlations between CSF sclerostin and global cognitive efficiency and cognitive domain scores remained significant, except for the positive trends with SCWT errors (*r* = 0.187; *p* = 0.064), and the negative trend with DS‐F score (*r* = −0.161; *p* = 0.082), supporting the robustness of the previous results.

The Bonferroni–Holm correction confirmed the statistical significance of the partial correlation results, apart from ROCF (corrected *p* = 0.058), DS‐B (corrected *p* = 0.068), and VFT–semantic scores (corrected *p* = 0.144).

All the correlations between CSF sclerostin levels and cognitive performance are summarized in Table 1, including both unadjusted analyses and partial correlations adjusted for age, sex, and education. For all cognitive domains, we considered *p* values uncorrected and corrected with the Bonferroni–Holm method to account for multiple comparisons.

**TABLE 1:** Correlations of CSF sclerostin levels and cognitive scores.

	Spearman correlation			Spearman correlation corrected for age, sex, and education		
	*r*	*p* value	Adjusted *p* value (Holm)	*r*	*p* value	Adjusted *p* value (Holm)
**Screening tests**						
MMSE	−0.304	**0.0003**		−0.244	**0.004**	
FAB	−0.358	**<0.0001**		−0.306	**<0.001**	
CDT	−0.257	**0.004**		−0.201	**0.027**	
**Memory**						
RAVLT immediate	−0.341	**0.0001**	**0.0004**	−0.268	**0.003**	**0.012**
RAVLT delayed	−0.328	**0.0002**	**0.0006**	−0.241	**0.008**	**0.024**
RAVLT recognition	−0.135	0.174	0.174	−0.071	0.483	0.483
ROCF	−0.430	**0.0028**	**0.0056**	−**0.333**	**0.029**	0.058
**Executive function**						
DS‐B	−0.262	**0.004**	**0.016**	−0.222	**0.017**	0.068
VFT–phonemic	−0.189	**0.032**	**0.064**	−0.176	**0.048**	0.144
TMT‐B	0.373	**0.0016**	**0.008**	**0.363**	**0.003**	**0.015**
SCWT Errors	0.258	**0.009**	**0.027**	0.187	0.064	0.144
SCWT	0.136	0.174	0.174	0.119	0.239	0.239
**Attention**						
DS‐F	−0.198	**0.030**	**0.030**	−0.161	0.082	0.082
TMT‐A	0.264	**0.006**	**0.012**	0.235	**0.017**	**0.034**
**Visuospatial abilities**						
Copy figure	−0.288	**0.003**	**0.003**	−0.275	**0.005**	**0.006**
VOSP Incomplete letters subtest	−0.309	**0.0007**	**0.0014**	−0.278	**0.003**	**0.006**
**Language**						
BNT	−0.192	**0.0295**	**0.0295**	−0.066	0.462	0.462
VFT–semantic	−0.264	**0.0032**	**0.0064**	−0.239	**0.009**	**0.018**

*Notes*: Correlations are expressed as Spearman correlation coefficient (*r*). For each cognitive domain, *p* values are shown both before and after Bonferroni–Holm correction for multiple comparisons. Spearman correlation coefficients (*r*) were adjusted for age, sex, and years of education in partial correlation analysis. Bold values highlight statistically significant correlations (*p* < 0.05).

Abbreviations: BNT, Boston Naming Test; CDT, Clock Drawing Test; CSF, cerebrospinal fluid; DS‐B, Digit Span Backward test; DS‐F, Digit Span Forward test; FAB, Frontal Assessment Battery; MMSE, Mini‐Mental State Examination; RAVLT, Rey Auditory Verbal Learning Test; ROCF, Rey–Osterrieth Complex Figure; SCWT, Stroop Color and Word Test; TMT‐A, Trail Making Test Part A; TMT‐B, Trail Making Test Part B; VFT–phonemic, Verbal Fluency Test—phonemic; VFT–semantic, Verbal Fluency Test –semantic; VOSP, Visual Object and Space Perception.

Sex‐stratified correlations between CSF sclerostin levels and neurocognitive scores are reported in Table 2. In women, all significant associations observed in the overall cohort were confirmed, except for ROCF (*r* = −0.333; *p* = 0.063) and TMT‐B (*r* = 0.263; *p* = 0.097), which showed a negative and a positive trend, respectively. In men, associations with CSF sclerostin levels were no longer statistically significant for FAB (*r* = −0.262; *p* = 0.075), CDT (*r* = −0.092; *p* = 0.532), DS‐B (*r* = −0.189; *p* = 0.217), VFT–phonemic (*r* = −0.074; *p* = 0.616), SCWT Errors (*r* = 0.219; *p* = 0.175), SCWT (*r* = 0.299; *p* = 0.060), DS‐F (*r* = −0.021; *p* = 0.892), Copy Figure (*r* = −0.241; *p* = 0.134), VOSP Incomplete letters subtest scores (*r* = −0.049; *p* = 0.754), BNT‐15 (*r* = −0.046; *p* = 0.752), and VFT–semantic (*r* = −0.249; *p* = 0.096).

**TABLE 2:** Correlations of CSF sclerostin levels and cognitive scores in women and men.

	Women			Men		
	Spearman correlation			Spearman correlation		
	r	*p* value	Adjusted *p* value (Holm)	r	*p* value	Adjusted *p* value (Holm)
**Screening tests**						
MMSE	−0.349	**0.001**		−0.272	**0.041**	0.123
FAB	−0.429	**<0.0001**		−0.262	0.075	0.150
CDT	−0.361	**0.0015**		−0.092	0.532	0.532
**Memory**						
RAVLT immediate	−0.303	**0.008**	**0.032**	−0.379	**0.009**	**0.027**
RAVLT delayed	−0.294	**0.009**	**0.027**	−0.408	**0.005**	**0.020**
RAVLT recognition	−0.155	0.222	0.222	−0.159	0.335	0.335
ROCF	−0.333	0.063	0.126	−0.617	**0.022**	**0.044**
**Executive functions**						
DS‐B	−0.308	**0.007**	**0.035**	−0.189	0.217	0.434
VFT–phonemic	−0.248	**0.026**	0.078	−0.074	0.616	0.616
TMT‐B	0.263	0.097	0.194	0.524	**0.004**	**0.020**
SCWT Errors	0.299	**0.018**	0.072	0.219	0.175	0.525
SCWT	0.021	0.873	0.873	0.299	0.060	0.240
**Attention**						
DS‐F	−0.319	**0.005**	**0.010**	−0.021	0.892	0.892
TMT‐A	0.259	**0.044**	**0.044**	0.314	**0.038**	0.076
**Visuospatial abilities**						
Copy figure	−0.319	**0.010**		−0.241	0.134	0.268
VOSP Incomplete letters subtest	−0.466	**<0.0001**		−0.049	0.754	0.754
**Language**						
BNT	−0.274	**0.015**	**0.030**	−0.046	0.752	0.752
VFT‐semantic	−0.265	**0.019**	**0.019**	−0.249	0.096	0.192

*Notes*: Correlations are expressed as Spearman correlation coefficient (*r*). For each cognitive domain, *p* values are shown both before and after Bonferroni–Holm correction for multiple comparisons. Spearman correlation coefficients (*r*) were adjusted for age, sex, and years of education in partial correlation analysis. Bold values highlight statistically significant correlations (*p* < 0.05).

Abbreviations: BNT, Boston Naming Test; CDT, Clock Drawing Test; CSF, cerebrospinal fluid; DS‐B, Digit Span Backward test; DS‐F, Digit Span Forward test; FAB, Frontal Assessment Battery; MMSE, Mini‐Mental State Examination; RAVLT, Rey Auditory Verbal Learning Test; ROCF, Rey–Osterrieth Complex Figure; SCWT, Stroop Color and Word Test; TMT‐A, Trail Making Test Part A; TMT‐B, Trail Making Test Part B; VFT–phonemic, Verbal Fluency Test—phonemic; VFT–semantic, Verbal Fluency Test –semantic; VOSP, Visual Object and Space Perception.

### 3.4 Comparison between low‐ and high‐level CSF sclerostin in patients with MCI and AD

Patients with MCI due to AD and AD were stratified based on their CSF sclerostin levels in low (below the median value) and high sclerostin (above the median value). Table 3 summarizes the results of this subgroup analysis. We found that patients with MCI due to AD with high sclerostin levels showed lower scores in MMSE (*p* = 0.006), FAB (*p* = 0.013), CDT (*p* = 0.034), RAVLT Immediate (*p* = 0.003), DS‐B (*p* = 0.009), VOSP Incomplete letters subtest (*p* = 0.009), and VFT–semantic scores (*p* = 0.018), while they had higher SCWT Errors (*p* = 0.019) and TMT‐A scores (0.032) compared to patients with MCI due to AD with low sclerostin levels. For AD, patients with high sclerostin levels showed higher scores in RAVLT (*p* = 0.039), while no difference was noted for all the other neuropsychological tests.

**TABLE 3:** Comparison between patient with low and high sclerostin levels.

	Patients with MCI			Patients with AD		
	Low CSF sclerostin	High CSF sclerostin	*p* value	Low CSF sclerostin	High CSF sclerostin	*p* value
**Screening tests**						
MMSE	23.91 ± 4.45	18.33 ± 5.41	**0.006** ^b^	16.11 ± 5.34	17.35 ± 5.04	0.311^a^
FAB	12.66 ± 3.62	9.11 ± 3.48	**0.013** ^b^	8.77 ± 3.47	8.69 ± 3.05	0.922^a^
CDT	9.18 ± 3.04	7.33 ± 2.18	**0.034** ^b^	5.75 ± 3.27	6.36 ± 3.13	0.506^b^
**Memory**						
RAVLT immediate	24.42 ± 7.93	15.44 ± 5.68	**0.003** ^a^	15.21 ± 8.29	15.57 ± 7.92	0.863^a^
RAVLT delayed	3.09 ± 2.71	1.22 ± 1.20	0.059^b^	0.61 ± 1.27	0.73 ± 1.44	0.978^b^
RAVLT recognition	0.84 ± 0.29	0.66 ± 0.17	0.159^b^	0.67 ± 0.15	0.73 ± 0.14	**0.039** ^b^
ROCF	30.85 ± 5.26	31.00 ± 1.41	0.762^b^	24.45 ± 6.49	19.31 ± 7.97	0.151^a^
**Executive function**						
DS‐B	3.23 ± 1.02	2.22 ± 0.97	**0.009** ^b^	2.27 ± 1.12	2.21 ± 1.14	0.777^b^
VFT–phonemic	19.76 ± 9.22	15.00 ± 8.72	0.17^a^	13.51 ± 10.61	13.97 ± 9.33	0.577^b^
TMT‐B	194.2 ± 117.1	287.5 ± 219.7	0.477^b^	296.9 ± 135.3	334.3 ± 128.3	0.517^a^
SCWT Errors	4.67 ± 7.94	9.06 ± 7.79	**0.019** ^b^	12.90 ± 9.11	10.26 ± 9.81	0.213^b^
SCWT	55.03 ± 84.60	67.56 ± 52.40	0.137^b^	40.55 ± 34.76	40.48 ± 28.09	0.994^a^
**Attention**						
DS‐F	5.16 ± 0.89	4.67 ± 1.23	0.15^b^	4.63 ± 0.97	4.63 ± 0.82	0.999^b^
TMT‐A	79.33 ± 40.54	111.5 ± 40.17	**0.032** ^b^	135.9 ± 82.21	109.2 ± 44.54	0.356^b^
**Visuospatial abilities**						
Copy figure	11.04 ± 3.19	9.33 ± 3.16	0.140^b^	8.08 ± 4.26	7.42 ± 3.29	0.311^b^
VOSP Incomplete letters subtest	17.39 ± 4.00	13.00 ± 5.81	**0.009** ^b^	12.30 ± 5.67	12.25 ± 6.46	0.859^b^
**Language**						
BNT	12.91 ± 2.18	11.67 ± 2.92	0.158^b^	9.89 ± 3.81	11.00 ± 2.70	0.349^b^
VFT–semantic	28.19 ± 7.59	20.00 ± 11.16	**0.018** ^a^	16.58 ± 9.69	18.86 ± 7.87	0.311^a^

*Notes*: Subgroup comparison in patients with MCI and AD stratified based on their CSF sclerostin levels in Low (under the median value) and High (over the median value). Bold values highlight statistically significant results (^a^ two‐tailed unpaired Student test or ^b^ Mann–Whitney test, *p* < 0.05).

Abbreviations: AD, Alzheimer's disease; BNT, Boston Naming Test; CDT, Clock Drawing Test; CSF, cerebrospinal fluid; DS‐B, Digit Span Backward test; DS‐F, Digit Span Forward test; FAB, Frontal Assessment Battery; MCI, mild cognitive impairment due to Alzheimer's disease; MMSE, Mini‐Mental State Examination; RAVLT, Rey Auditory Verbal Learning Test; ROCF, Rey–Osterrieth Complex Figure; SCWT, Stroop Color and Word Test; TMT‐A, Trail Making Test Part A; TMT‐B, Trail Making Test Part B; VFT–phonemic, Verbal Fluency Test—phonemic; VFT‐semantic, Verbal Fluency Test–semantic; VOSP, Visual Object and Space Perception.

## 4 DISCUSSION

In this study, we explored the association between CSF sclerostin, an inhibitor of the Wnt‐β catenin signaling pathway, and neurocognitive performance, both global and domain specific, in the AD continuum. Notably, we observed that higher CSF sclerostin levels were associated with neurocognitive dysfunctions in all the cognitive domains under investigation. This finding was particularly evident in the prodromal phase of AD, that is, MCI due to AD; indeed, significant differences in neurocognitive performance emerged between patients with MCI due to AD with low and high CSF sclerostin levels.

AD and osteoporosis are both age‐related disease for which aging is a relevant risk factor.^30^ Several epidemiological studies and meta‐analyses have shown an association between AD and osteoporosis, so that several research studies have tried to identify shared molecular pathways involved in their pathogenesis.^30^

The Wnt‐β catenin signaling is a crucial pathway for multiple processes in bones and the brain.^31^ In bone tissue, Wnt‐β catenin signaling regulates skeletal homeostasis and its activation leads to increased bone formation,^31^ while in the nervous system, this pathway is critical for neuronal survival, neurogenesis, synaptic plasticity, and so on.^31^ Of note, the dysregulation of the Wnt‐β catenin pathway has been documented in the brain of patients with AD and in mouse models of AD both showing memory deficits.^32^ The osteocyte‐derived sclerostin, a potent Wnt/*β* catenin pathway inhibitor, has emerged as a potential mediator of AD pathophysiology. Indeed, elevated plasma sclerostin levels have been associated with increased brain Aβ burden in cognitively normal older adults at high risk of AD,^33^ and, very recently, sclerostin has been proposed as a potential candidate of cognitive impairment in AD.^10^

In line with this pivotal study that demonstrated the negative correlation between serum sclerostin levels and MMSE score in old subjects and patients with AD,^10^ our results reinforced the association between higher CSF sclerostin levels and lower MMSE scores in the continuum of AD. Moreover, on stratifying all patients by MMSE score, we found increased CSF sclerostin levels in patients with moderate cognitive impairment; therefore, it is plausible to suppose that sclerostin could be involved in the early stages of clinical AD.

As a novelty, we found a negative correlation between CSF sclerostin levels and memory test scores, mainly with immediate and delayed RAVLT scores, but not with RAVLT and ROCF recognition scores, suggesting a selective involvement of sclerostin on specific brain areas that requires further investigation. Indeed, perirhinal and entorhinal cortices play a crucial role in familiarity‐based recognition memory, a process often impaired in the early stages of AD. These medial temporal lobe structures are among the first regions to exhibit neurofibrillary pathology and atrophy in AD, disrupting the neural circuits that support the rapid, context‐free identification of previously encountered stimuli. Mounting evidence suggests that degeneration in these areas contributes significantly to the decline in familiarity processing, distinguishing it from deficits in recollection, which are more strongly associated with hippocampal damage.^34^ Our findings confirmed, in humans, what previously has been observed in transgenic mice and mouse models of AD both overexpressing the sclerostin gene.^10^ Indeed, it has been demonstrated that higher serum sclerostin levels were associated with spatial memory deficit in the Morris Water Maze test, and memory impairments for novel object recognition, due to synaptic plasticity dysregulation and increased brain Aβ deposition.^10^

Besides memory, this study provides novel and compelling evidence that elevated CSF sclerostin levels are not only linked to memory decline but are also broadly associated with impairments across multiple cognitive domains usually compromised in AD. The observed correlations with executive function, attention, visuospatial abilities, and language suggest a more extensive role of sclerostin in AD‐related cognitive dysfunction than previously recognized. Specifically, we observed that CSF sclerostin primarily correlated with TMT‐B score for the executive functions, with TMT‐A score for attention, with Copy Figure and VOSP incomplete letter subtest scores for visuospatial abilities, and VFT–semantic score for language.

Of note, we found that the association between CSF sclerostin and cognitive impairment was prominent in patients with MCI due to AD, further evidencing the impact of sclerostin on cognition in the early AD stages. These findings are in line with the results of our previous investigation showing the correlation of CSF sclerostin with Aβ42, tau pathology, and dementia severity in the early stages of clinical AD.^11^ However, ROC analyses evidenced a limited discriminative performance of CSF sclerostin across clinical groups suggesting that the osteokine reflects disease‐related biological processes but has limited capacity for individual‐level classification. Thus, sclerostin's clinical utility is likely to be limited as a stand‐alone marker, although it may be useful as a supportive measure when combined with other biomarkers.

Interestingly, sex‐stratified analyses showed that the association between CSF sclerostin levels and cognitive performance was maintained in women but not in men. Although these findings should be interpreted cautiously, they may point to sex‐dependent mechanisms linking sclerostin biology and cognitive function. Further investigations are warranted to clarify the molecular pathways involved, as well as the potential contribution of sex hormones in explaining these differences.

We are aware that our study has some limitations. First, this is a cross‐sectional study that did not allow us to determine a clear cause‐and‐effect relationship between sclerostin and cognitive decline in the AD continuum, although it will be helpful for future longitudinal investigations on this topic.

Moreover, our results on CSF samples need to be extended to serum and/or plasma samples to find a more suitable biological sample for easier diagnosis. Future research integrating neuroimaging techniques will be essential to map the brain regions linked to elevated CSF sclerostin levels and to better understand how this biomarker relates to structural and functional changes underlying cognitive impairment.

Overall, our findings evidenced that the correlation between sclerostin levels and MMSE was not limited to patients with AD but can be extended to the clinical stages of AD. Moreover, sclerostin negatively impacted all the cognitive domains, particularly in the early stages of clinical AD, and this effect remained significant even after correcting for certain relevant variables in AD.^35^

Finally, the association between sclerostin and neurocognitive impairment could be of great clinical importance for the treatment of AD if additional investigations substantiate the role of sclerostin in the pathogenesis of AD.

## AUTHOR CONTRIBUTIONS

**Patrizia Pignataro**: Writing – original draft; investigation; data curation; formal analysis. **Manuela Dicarlo**: Writing – original draft; investigation; data curation; formal analysis. **Daniele Urso**: Writing – original draft; investigation; data curation; formal analysis. **Chiara Zecca**: Writing – original draft; investigation; data curation; formal analysis. **Graziana Colaianni**: Investigation; data curation; formal analysis. **Maria Teresa Dell'Abate**: Writing – original draft; investigation; data curation; formal analysis. **Davide Vilella**: Writing – original draft; investigation; data curation. **Francesco Borlizzi**: Investigation; data curation; formal analysis. **Clelia Suriano**: Investigation; data curation; formal analysis. **Angela Oranger**: Investigation; data curation; formal analysis. **Silvia Colucci**: Writing review & editing; validation; data curation; formal analysis. **Maria Grano**: Writing review & editing; validation; data curation; formal analysis; funding acquisition. **Giancarlo Logroscino**: Writing review & editing; validation; formal analysis; funding acquisition.

## CONFLICT OF INTEREST STATEMENT

The authors declare no conflicts of interest. Author disclosures are available in the Supporting Information.

## CONSENT STATEMENT

All participants enrolled in this study provided their written informed consent.

## Supporting information



Supporting Information

Supporting Information

Supporting Information

Supporting Information

Supporting Information

Supporting Information

Supporting Information

Supporting Information

Supporting Information

Supporting Information

Supporting Information
