# Supplementary material for: Elevated sclerostin levels in cerebrospinal fluid are associated with cognitive impairment in the Alzheimer's disease continuum
Source: Alzheimers Dement (Amst). 2026 Jun 30;18(3):e70417. doi: 10.1002/dad2.70417 (PMC13319414; doi:10.1002/dad2.70417)
Supplement: Supplementary file 1 — Supporting Information [file DAD2-18-e70417-s002.docx]

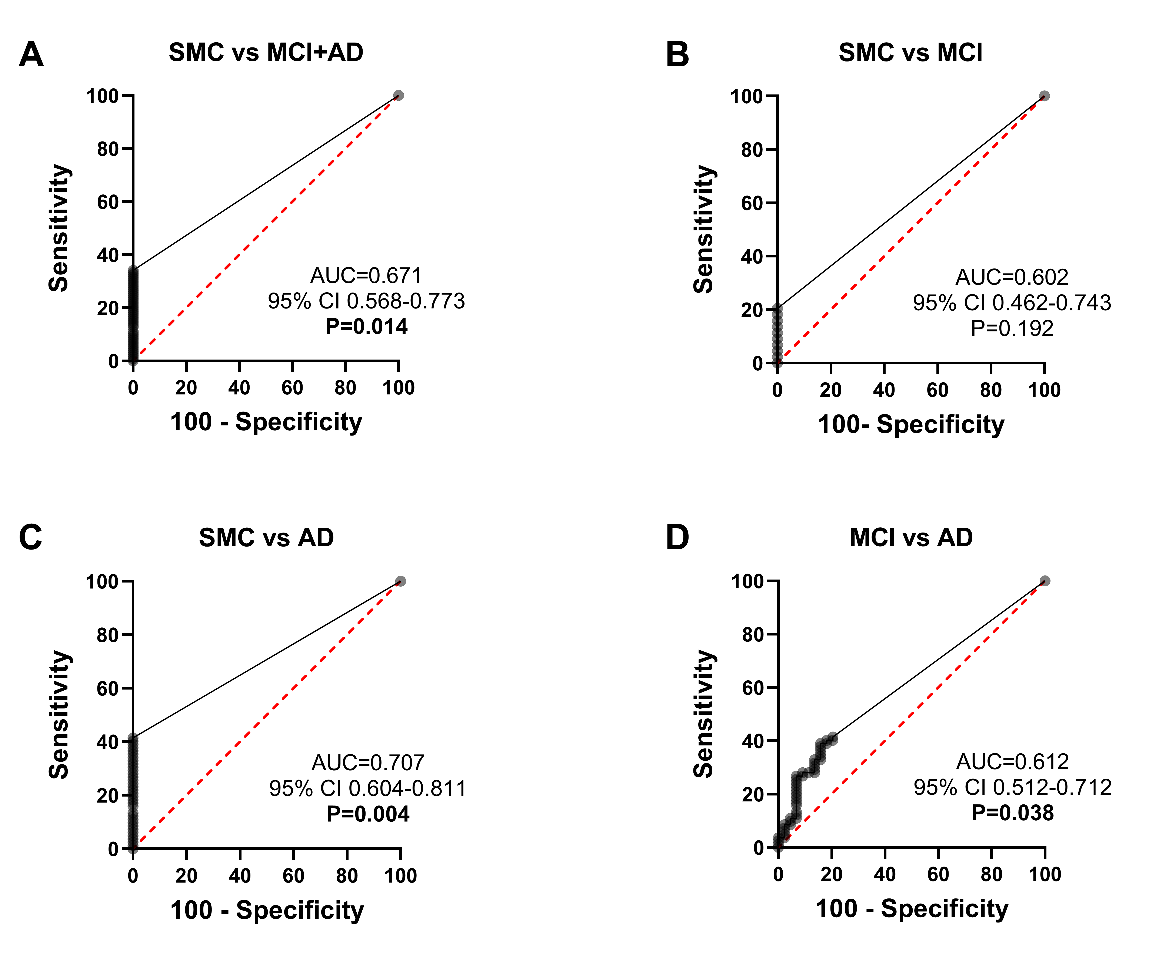


**Figure S1***.* Receiver operating characteristic (ROC) curves of CSF sclerostin for the discrimination between different patient subgroups. A) Cognitively normal subjects with subjective memory complaints (SMC) vs cognitively impaired patients (MCI due to AD + AD); B) SMC vs MCI due to AD; C) SMC vs AD, and D) MCI due to AD vs AD. For each comparison, the area under the curve (AUC), 95% confidence interval (95% CI), and P values are reported. The dashed red diagonal lines indicate the performance of a non-informative classifier (AUC=0.5). Bold values indicate statistically significant results (P < 0.05).

Abbreviations: MCI, mild cognitive impairment due to AD; AD, Alzheimer's dementia.
