# Supplementary material for: Elevated sclerostin levels in cerebrospinal fluid are associated with cognitive impairment in the Alzheimer's disease continuum
Source: Alzheimers Dement (Amst). 2026 Jun 30;18(3):e70417. doi: 10.1002/dad2.70417 (PMC13319414; doi:10.1002/dad2.70417)
Supplement: Supplementary file 3 — Supporting Information [file DAD2-18-e70417-s010.docx]

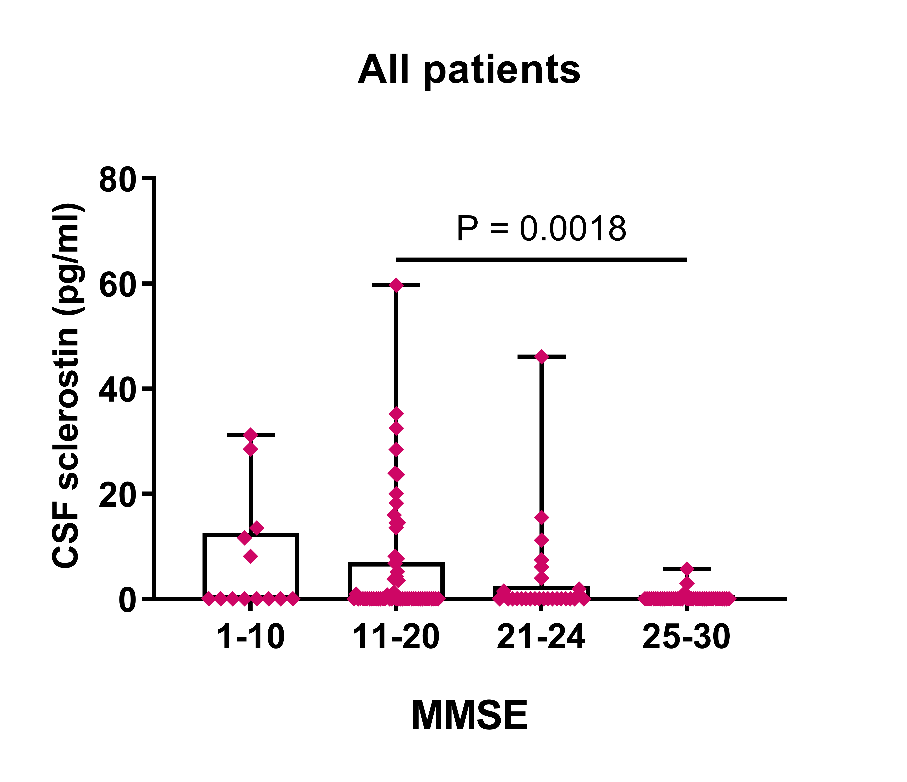


**Figure S3.** Comparison of CSF sclerostin levels in patients with severe (MMSE: 1-10), moderate (MMSE: 11-20), and mild cognitive impairment (MMSE: 21-24), and cognitively normal subjects (MMSE: 25-30). Data are presented as box-and-whisker with median and interquartile ranges, from max to min, with all data points shown. Horizontal bars show significant differences among groups (Kruskal-Wallis test/Dunn's multiple comparison test, P < 0.05).

Abbreviations: CSF, cerebrospinal fluid.
