# Supplementary material for: Elevated sclerostin levels in cerebrospinal fluid are associated with cognitive impairment in the Alzheimer's disease continuum
Source: Alzheimers Dement (Amst). 2026 Jun 30;18(3):e70417. doi: 10.1002/dad2.70417 (PMC13319414; doi:10.1002/dad2.70417)
Supplement: Supplementary file 5 — Supporting Information [file DAD2-18-e70417-s004.docx]

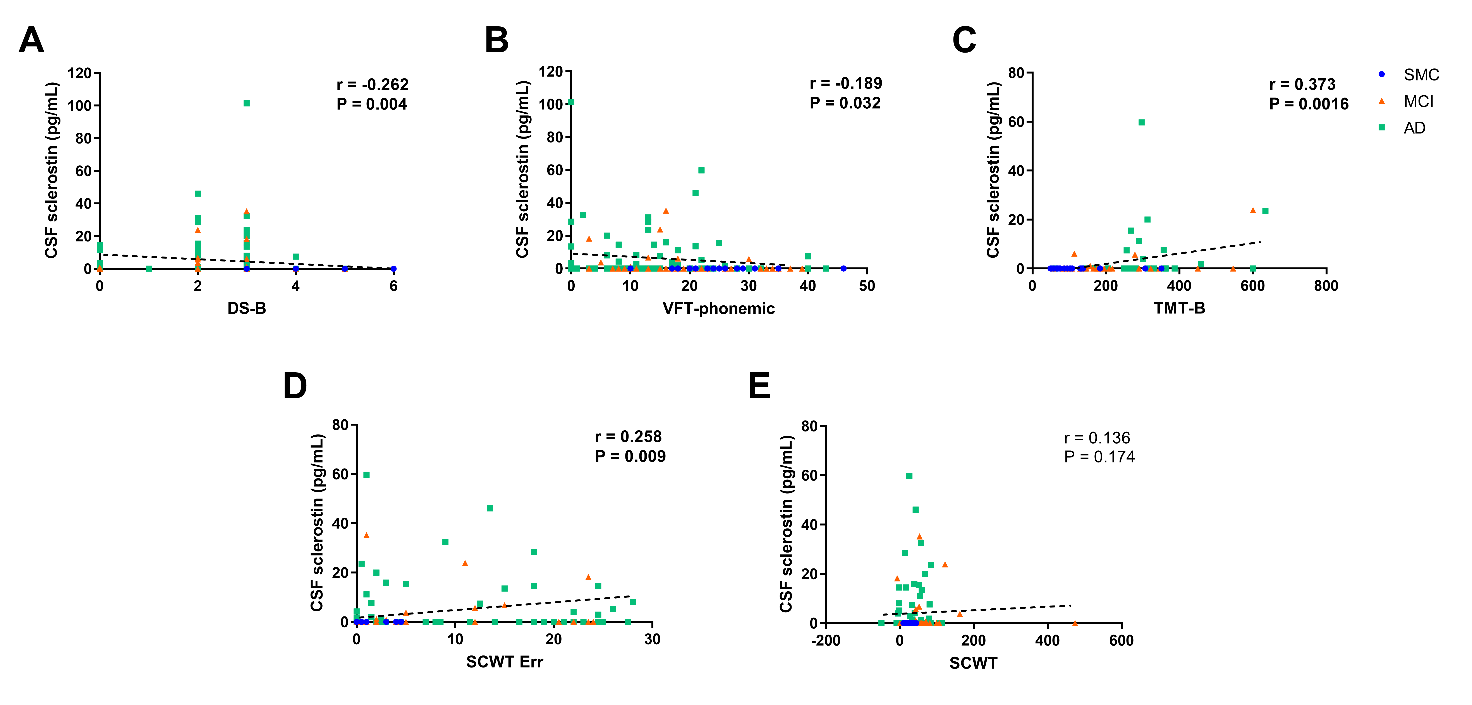


**Figure S5***.* Correlations between CSF sclerostin levels and executive function test scores. A) Negative correlation with DS-B, and B) VFT-phonemic. C) Positive correlation with TMT-B, and D) SCWT Err. E) Positive trend with SCWT.

Dotted lines represent Spearman linear regressions (r and P values as indicated). Bold values highlight statistically significant correlations.

Abbreviations: SMC, subjective memory complaints; MCI, mild cognitive impairment due to AD; AD, Alzheimer’s dementia; CSF, cerebrospinal fluid; DS-B, Digit Span Backward test; VFT, Verbal Fluency Test; TMT-B, Trail Making Test Version B; SCWT, Stroop Color and Word Test; SCWT Err, Stroop Color and Word Test Errors.
