# Supplementary material for: Elevated sclerostin levels in cerebrospinal fluid are associated with cognitive impairment in the Alzheimer's disease continuum
Source: Alzheimers Dement (Amst). 2026 Jun 30;18(3):e70417. doi: 10.1002/dad2.70417 (PMC13319414; doi:10.1002/dad2.70417)
Supplement: Supplementary file 7 — Supporting Information [file DAD2-18-e70417-s009.docx]

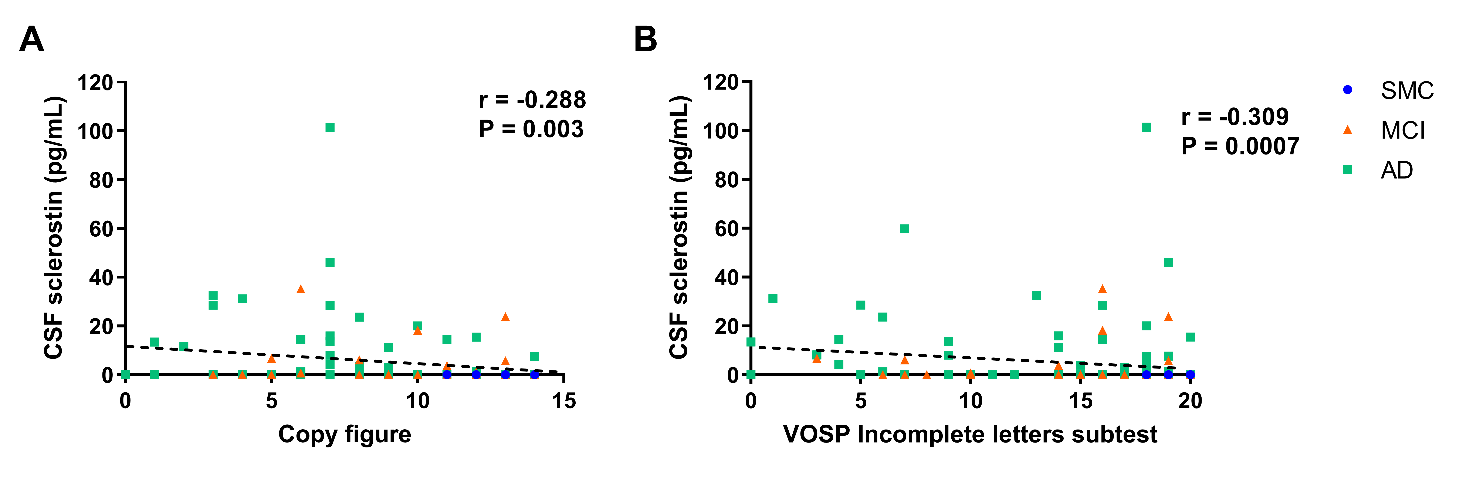


**Figure S7***.* Correlations between CSF sclerostin levels and visuospatial abilities’s test scores. A) Negative correlation with Copy Figure, and B) VOSP Incomplete letters subtest. Dotted lines represent Spearman linear regressions (r and P values as indicated). Bold values highlight statistically significant correlations.

Abbreviations: SMC, subjective memory complaints; MCI, mild cognitive impairment due to AD; AD, Alzheimer’s dementia; CSF, cerebrospinal fluid; VOSP, Visual Object and Space Perception.
