# Supplementary material for: Elevated sclerostin levels in cerebrospinal fluid are associated with cognitive impairment in the Alzheimer's disease continuum
Source: Alzheimers Dement (Amst). 2026 Jun 30;18(3):e70417. doi: 10.1002/dad2.70417 (PMC13319414; doi:10.1002/dad2.70417)
Supplement: Supplementary file 9 — Supporting Information [file DAD2-18-e70417-s003.docx]

**SUPPLEMENTARY TABLE S1.** Cognitive domains and neuropsychological tests administered to the study participants.

|  | **Neuropsychological Tests** |
| --- | --- |
| **Screening tests** |  |
|  | Mini-Mental Status Examination (MMSE)  Frontal Assessment Battery (FAB)  Clock Drawing Test (CDT) |
| **Cognitive domains** |  |
| Memory | Rey Auditory Verbal Learning Test (RAVLT)   - Immediate - Delayed - Recognition   Rey–Osterrieth Complex Figure (ROCF) |
| Executive functions | Digit Span- Backward (DS-B)  Verbal Fluency Test (VFT) - Phonemic  Trail Making Test version B (TMT-B)  Stroop Color and Word Test (SCWT)   - Stroop Color and Word Test (SCWT) - Stroop Color and Word Test (SCWT) Error |
| Attention | Digit Span - Forward (DS-F)  Trail Making Test version A (TMT-A) |
| Visuospatial abilities | Copy figure  Visual Object and Space Perception (VOSP) incomplete letters subtest |
| Language | Boston Naming Test (BNT) – short version  Verbal Fluency Test (VFT) - Semantic |
