# Supplementary material for: Elevated sclerostin levels in cerebrospinal fluid are associated with cognitive impairment in the Alzheimer's disease continuum
Source: Alzheimers Dement (Amst). 2026 Jun 30;18(3):e70417. doi: 10.1002/dad2.70417 (PMC13319414; doi:10.1002/dad2.70417)
Supplement: Supplementary file 10 — Supporting Information [file DAD2-18-e70417-s006.docx]

**SUPPLEMENTARY TABLE S2.** Normality assessment of continuous variables.

|  | Variable | Group | W | P value | Distribution |
| --- | --- | --- | --- | --- | --- |
| Demographic and clinical data | Age | SMC | 0.956 | 0.468 | Normal |
|  |  | MCI | 0.985 | 0.817 | Normal |
|  |  | AD | 0.985 | 0.479 | Normal |
|  | Education | SMC | 0.921 | 0.103 | Normal |
|  |  | MCI | 0.873 | **0.0002** | Non-normal |
|  |  | AD | 0.894 | **<0.0001** | Non-normal |
|  | CDR total | SMC | 0.244 | **<0.0001** | Non-normal |
|  |  | MCI | 0.273 | **<0.0001** | Non-normal |
|  |  | AD | 0.786 | **<0.0001** | Non-normal |
|  | CDR-SOB | SMC | 0.728 | **0.0005** | Non-normal |
|  |  | MCI | 0.973 | 0.470 | Normal |
|  |  | AD | 0.859 | **<0.0001** | Non-normal |
|  | CSF sclerostin | All patients | 0.425 | **<0.0001** | Non-normal |
|  | CSF sclerostin | Women | 0.427 | **<0.0001** | Non-normal |
|  | CSF sclerostin | Men | 0.489 | **<0.0001** | Non-normal |
| Screening test | MMSE | SMC | 0.897 | **0.035** | Non-normal |
|  |  | MCI | 0.898 | **0.001** | Non-normal |
|  |  | AD | 0.974 | 0.118 | Normal |
|  | FAB | SMC | 0.822 | **0.004** | Non-normal |
|  |  | MCI | 0.936 | **0.023** | Non-normal |
|  |  | AD | 0.980 | 0.347 | Normal |
|  | CDT | SMC | 0.740 | **0.0002** | Non-normal |
|  |  | MCI | 0.918 | **0.005** | Non-normal |
|  |  | AD | 0.964 | 0.059 | Normal |
| Memory | RAVLT immediate | SMC | 0.856 | **0.011** | Non-normal |
|  |  | MCI | 0.979 | 0.633 | Normal |
|  |  | AD | 0.975 | 0.223 | Normal |
|  | RAVLT Delayed | SMC | 0.896 | **0.048** | Non-normal |
|  |  | MCI | 0.886 | **0.0006** | Non-normal |
|  |  | AD | 0,567 | **<0.0001** | Non-normal |
|  | RAVLT recognition | SMC | 0.700 | **<0.0001** | Non-normal |
|  |  | MCI | 0.701 | **<0.0001** | Non-normal |
|  |  | AD | 0.946 | **0.026** | Non-normal |
|  | ROCF | SMC | 0.832 | **0.017** | Non-normal |
|  |  | MCI | 0.844 | **0.014** | Non-normal |
|  |  | AD | 0.946 | 0.359 | Normal |
| Executive functions | DS-B | SMC | 0.845 | **0.007** | Non-normal |
|  |  | MCI | 0.882 | **0.0006** | Non-normal |
|  |  | AD | 0.858 | **<0.0001** | Non-normal |
|  | VFT-phonemic | SMC | 0.947 | 0.375 | Normal |
|  |  | MCI | 0.971 | 0.339 | Normal |
|  |  | AD | 0.939 | **0.003** | Non-normal |
|  | TMT-B | SMC | 0.741 | **0.0002** | Non-normal |
|  |  | MCI | 0.818 | **0.0002** | Non-normal |
|  |  | AD | 0.925 | 0.097 | Normal |
|  | SCWT | SMC | 0.945 | 0.352 | Normal |
|  |  | MCI | 0.526 | **<0.0001** | Non-normal |
|  |  | AD | 0.988 | 0.891 | Normal |
|  | SCWT Err | SMC | 0.637 | **<0.0001** | Non-normal |
|  |  | MCI | 0.719 | **<0.0001** | Non-normal |
|  |  | AD | 0.888 | **0.0003** | Non-normal |
| Attention | DS-F | SMC | 0.847 | **0.008** | Non-normal |
|  |  | MCI | 0.889 | **0.0009** | Non-normal |
|  |  | AD | 0.860 | **<0.0001** | Non-normal |
|  | TMT-A | SMC | 0.749 | **0.0003** | Non-normal |
|  |  | MCI | 0.872 | **0.0004** | Non-normal |
|  |  | AD | 0.803 | **<0.0001** | Non-normal |
| Visuospatial abilities | Copy figure | SMC | 0.846 | 0.068 | Normal |
|  |  | MCI | 0.862 | **0.0006** | Non-normal |
|  |  | AD | 0.954 | **0.021** | Non-normal |
|  | VOSP Incomplete letters subtest | SMC | 0.738 | **0.0005** | Non-normal |
|  |  | MCI | 0.747 | **<0.0001** | Non-normal |
|  |  | AD | 0.902 | **0.0001** | Non-normal |
| Language | BNT | SMC | 0.831 | **0.004** | Non-normal |
|  |  | MCI | 0.858 | **<0.0001** | Non-normal |
|  |  | AD | 0.920 | **0.0003** | Non-normal |
|  | VFT-semantic | SMC | 0.969 | 0.769 | Normal |
|  |  | MCI | 0.969 | 0.335 | Normal |
|  |  | AD | 0.967 | 0.083 | Normal |

Notes: Shapiro–Wilk test results are expressed as W statistics (W) and corresponding P values. W values closer to 1 indicated greater conformity to a normal distribution. Bold values highlight a significant deviation from normality (P < 0.05). Abbreviations: SMC, subjective memory complaints; MCI, mild cognitive impairment due to AD; AD, Alzheimer’s dementia; MMSE, Mini-Mental Status Examination; FAB, Frontal Assessment Battery; CDT, Clock Drawing Test; RAVLT, Rey Auditory Verbal Learning Test; ROCF, Rey–Osterrieth Complex Figure; DS-B, Digit Span Backward test; VFT - semantic, Verbal Fluency Test - semantic; VFT- phonemic, Verbal Fluency Test - phonemic; TMT-B, Trail Making Test Version B; SCWT, Stroop Color and Word Test; DS-F, Digit Span Forward; TMT-A, Trail Making Test version A; VOSP, Visual Object and Space Perception; BNT, Boston Naming Test.
