# Supplementary material for: Elevated sclerostin levels in cerebrospinal fluid are associated with cognitive impairment in the Alzheimer's disease continuum
Source: Alzheimers Dement (Amst). 2026 Jun 30;18(3):e70417. doi: 10.1002/dad2.70417 (PMC13319414; doi:10.1002/dad2.70417)
Supplement: Supplementary file 11 — Supporting Information [file DAD2-18-e70417-s011.docx]

**SUPPLEMENTARY TABLE S3**. Cognitive scores in SMC, MCI and AD patients

| Cognitive parameter | SMC | MCI | AD | *P* value | SMC vs MCI | SMC vs AD | MCI vs AD |
| --- | --- | --- | --- | --- | --- | --- | --- |
| *Screening tests* | | | | | | | |
| MMSE | 27.85±1.87;  28 (26.25-29) | 22.74±5.14;  24 (20-26) | 16.62±5.22;  17 (14-20) | **<0.0001**^b^ | **0.0034** | **<0.0001** | **<0.0001** |
| FAB | 15.94±2.36;  16 (14.5-18) | 11.88±3.84;  12 (9.5-15) | 8.74±3.27;  9 (6-11) | **<0.0001**^b^ | **0.0038** | **<0.0001** | **0.0003** |
| CDT | 11.39±1.61;  12 (11-12) | 8.79±2.95;  9 (7.75-11) | 6.02±3.2;  5.5 (4-8) | **<0.0001**^b^ | **0.0149** | **<0.0001** | **0.0003** |
| *Memory* | | | | | | | |
| RAVLT Immediate | 36.44±6.72;  37.5 (34.8-42) | 22.50±8.33;  22 (17-28.25) | 15.38±8.05;  15 (10-20) | **<0.0001**^b^ | **0.0004** | **<0.0001** | **0.0010** |
| RAVLT  Delayed | 6.5±2.15;  7 (5.75-8) | 2.69±2.57;  2 (0.75-4.25) | 0.67±1.34;  0 (0-1) | **<0.0001**^b^ | **0.0008** | **<0.0001** | **<0.0001** |
| RAVLT  Recognition | 1.12±0.47;  0.93 (0.88-1.23) | 0.82±0.29;  0.77 (0.69-0.86) | 0.70±0.15;  0.69 (0.60-0.78) | **<0.0001**^b^ | **0.0032** | **<0.0001** | 0.0741 |
| ROCF | 33.15±3.21;  34 (32-36) | 30.87±4.88;  32 (30-34) | 22.17±7.44;  22 (16.38-28) | **<0.0001**^b^ | 0.7197 | **<0.0001** | **0.0060** |
| *Executive functions* | | | | | | | |
| DS-B | 3.94±0.94;  4 (3-5) | 3±1.09;  3 (2-4) | 2.25±1.12;  2 (2-3) | **<0.0001**^b^ | **0.0250** | **<0.0001** | **0.0026** |
| VFT-phonemic | 25.17±7.78;  25 (20-29) | 18.77±9.22;  18 (13-25) | 13.71±10.01;  12.5 (7-20.75) | **<0.0001**^b^ | 0.0524 | **<0.0001** | **0.0186** |
| TMT-B | 119.4±84.32;  91 (68-134.3) | 207.1±134.2;  177 (110.5-248) | 313.9±130.4;  298.5 (254.5-360.8) | **<0.0001**^b^ | **0.0316** | **<0.0001** | **0.0161** |
| SCWT | 26.65±10.49;  28.75 (17.38-33.13) | 57.74±78.29;  39.5 (23.48-61) | 40.51±31.3;  40 (21-59) | **0.0450**^b^ | 0.0524 | 0.0852 | >0.999 |
| SCWT (Error) | 0.83±1.46;  0 (0-1) | 5.62±8.01;  1 (0-11.5) | 11.61±9.45;  9 (2.5-21) | **<0.0001**^b^ | 0.0912 | **<0.0001** | **0.0011** |
| *Attention* | | | | | | | |
| DS-F | 5.72±0.75;  6 (5-6) | 5.05±0.99;  5 (4-6) | 4.63±0.91;  4.5 (4-5) | **<0.0001**^b^ | **0.0333** | **<0.0001** | 0.1563 |
| TMT-A | 43.63±18.87;  37 (32.78-48.25) | 84.28±41.64;  76 (62-99) | 124.2±69;  104 (76.25-158.5) | **<0.0001**^b^ | **0.0006** | **<0.0001** | **0.0061** |
| *Visuospatial abilities* | | | | | | | |
| Copy figure | 12.89±0.93;  13 (12.5-13.5) | 10.58±3.23;  12 (8.5-13) | 7.82±3.90;  8 (5-11) | **<0.0001**^b^ | 0.1672 | **0.0002** | **0.0023** |
| VOSP Incomplete letters subtest | 19.44±0.73;  20 (19-20) | 16.54±4.67;  19 (15.5-20) | 12.28±5.99;  14 (7-18) | **<0.0001**^b^ | 0.0633 | **<0.0001** | **<0.0001** |
| *Language* | | | | | | | |
| BNT | 13.94±1.16;  14 (13-15) | 12.65±2.37;  13 (11-14) | 10.38±3.39;  11 (8.25-13) | **<0.0001**^b^ | 0.1765 | **<0.0001** | **0.0005** |
| VFT-semantic | 34.39±6.57;  33 (30-39.5) | 26.55±8.89;  27.5 (20-34) | 17.60±8.94;  18 (10.5-24.5) | **<0.0001**^a^ | **0.0049** | **<0.0001** | **<0.0001** |

Notes: Values are presented as mean ± SD; Median (Interquartile range Q1-Q3). Notes: Bold values highlight statistically significant differences among patient groups (^a^ ANOVA-Tukey test and ^b^ Kruskal-Wallis-Dunn’s test, *P* < 0.05).

Abbreviations: SMC, subjective memory complaints; MCI, mild cognitive impairment due to AD; AD, Alzheimer’s dementia; MMSE, Mini-Mental Status Examination; FAB, Frontal Assessment Battery; CDT, Clock Drawing Test; RAVLT, Rey Auditory Verbal Learning Test; ROCF, Rey–Osterrieth Complex Figure; DS-B, Digit Span Backward test; VFT - semantic, Verbal Fluency Test - semantic; VFT- phonemic, Verbal Fluency Test - phonemic; TMT-B, Trail Making Test Version B; SCWT, Stroop Color and Word Test; DS-F, Digit Span Forward; TMT-A, Trail Making Test version A; VOSP, Visual Object and Space Perception; BNT, Boston Naming Test; SD, standard deviation; Q1, lower quartile; Q3, upper quartile.
